# Energy Associated With Dynamic Network Changes in Patients With Multiple Sclerosis and Cognitive Impairment

**DOI:** 10.1212/WNL.0000000000209952

**Published:** 2024-10-11

**Authors:** Tommy A.A. Broeders, Maureen van Dam, Giuseppe Pontillo, Vasco Rauh, Linda Douw, Ysbrand D. van der Werf, Joep Killestein, Frederik Barkhof, Christiaan H. Vinkers, Menno M. Schoonheim

**Affiliations:** From the MS Center Amsterdam (T.A.A.B., M.v.D., V.R., L.D., Y.D.v.d.W., C.H.V., M.M.S.), Anatomy & Neurosciences, and MS Center Amsterdam (G.P., F.B.), Radiology and Nuclear Medicine, Vrije Universiteit Amsterdam, Amsterdam Neuroscience, Amsterdam UMC location VUmc, the Netherlands; Queen Square Institute of Neurology and Centre for Medical Image Computing (G.P., F.B.), University College London, United Kingdom; Departments of Advanced Biomedical Sciences and Electrical Engineering and Information Technology (G.P.), University of Naples “Federico II,” Italy; MS Center Amsterdam (J.K.), Neurology, and MS Center Amsterdam (C.H.V.), Psychiatry, Vrije Universiteit Amsterdam, Amsterdam Neuroscience, Amsterdam UMC location VUmc; Amsterdam Public Health (C.H.V.), Mental Health Program; and GGZ inGeest Mental Health Care (C.H.V.), Amsterdam, the Netherlands.

## Abstract

**Background and Objectives:**

Patients with multiple sclerosis (MS) often experience cognitive impairment, and this is related to structural disconnection and subsequent functional reorganization. It is unclear how specific patterns of functional reorganization might make it harder for cognitively impaired (CI) patients with MS to dynamically adapt how brain regions communicate, which is crucial for normal cognition. We aimed to identify dynamic functional network patterns that are relevant to cognitive impairment in MS and investigate whether these patterns can be explained by altered energy costs.

**Methods:**

Resting-state functional and diffusion MRI was acquired in a cross-sectional design, as part of the Amsterdam MS cohort. Patients with clinically definitive MS (relapse-free) were classified as CI (≥2/7 domains *Z* < −2), mildly CI (MCI) (≥2/7 domains *Z* < −1.5), or cognitively preserved (CP) based on an expanded Brief Repeatable Battery of Neuropsychological Tests. Functional connectivity states were determined using *k*-means clustering of moment-to-moment cofluctuations (i.e., edge time series), and the resulting state sequence was used to characterize the frequency of transitions. Control energy of the state transitions was calculated using the structural network with network control theory.

**Results:**

Imaging and cognitive data were available for 95 controls and 330 patients (disease duration: 15 years; 179 CP, 65 MCI, and 86 CI). We identified a “visual network state,” “sensorimotor network state,” “ventral attention network state,” and “default mode network state.” CI patients transitioned less frequently between connectivity states compared with CP (β = −5.78; *p* = 0.038). Relative to the time spent in a state, CI patients transitioned less from a “default mode network state” to a “visual network state” (β = −0.02; *p* = 0.004). The CI patients required more control energy to transition between states (β = 0.32; *p* = 0.007), particularly for the same transition (β = 0.34; *p* = 0.049).

**Discussion:**

This study showed that it costs more energy for MS patients with cognitive impairment to dynamically change the functional network, possibly explaining why these transitions occur less frequently. In particular, transitions from a default mode network state to a visual network state were relevant for cognition in these patients. To further study the order of events leading to these network disturbances, future work should include longitudinal data across different disease stages.

## Introduction

Cognitive impairment is a highly debilitating symptom of multiple sclerosis (MS) that occurs in up to 65% of patients.^1^ In MS, neurodegeneration and neuroinflammation damage the CNS, giving rise to focal lesions and brain atrophy.^2^ These patterns can be detected using MRI, which is essential for diagnosis,^3^ but do not fully explain clinical outcomes such as cognitive impairment.^4^ In MS, damage to the anatomical pathways between brain regions (i.e., structural connectivity) can also affect their communication (i.e., functional connectivity).^5^ Novel conceptual and mathematical tools were needed to describe how the MS brain is (dis)organized and how this may lead to cognitive impairment. In this push, network neuroscience has been crucial in which the brain is represented as a graph consisting of brain regions (i.e., nodes) that are structurally and functionally connected (i.e., edges).^6^ This is a more holistic approach because it does not focus on single connections but models how all regions interact. Using this framework, it was learned that functional reorganization likely plays an important role in compensating for structural damage in the early stages of MS, which in theory would be energetically costly.^7^ As structural damage accumulates, a critical threshold is passed after which the network cannot function properly and costly compensatory strategies fail.^8^ Key to this loss of function seems to be the overload of a few highly connected brain regions (i.e., hubs), such as regions in the default-mode network (DMN).^9^

A hub overload could leave the brain network less dynamically adaptable to cognitive challenges.^7^ However, these dynamic characteristics have often not been explicitly analyzed because most earlier studies averaged connectivity over time (i.e., static connectivity).^10^ This means that time-dependent patterns (i.e., dynamic connectivity) were neglected. Dynamic network alterations are integral brain processes relevant to cognitive functioning by themselves, for instance allowing the brain to transition between modes of segregated and integrated processing.^11^ Recent studies have observed disturbed network dynamics in MS patients with cognitive impairment, even without subjecting them to an explicit task (i.e., resting-state). For example, regions in the DMN, frontoparietal network (FPN), and visual network (VIS) showed less connectivity dynamics.^12^ This has been interpreted as indicating that hubs can be stuck in an “overloaded” state.^7^ Brain network dynamics of cognitively impaired (CI) patients with MS might be affected in nonhubs as well,^13^ so it is important to look at dynamics of the functional network as a whole. Accordingly, recent studies applied a holistic model in which recurrent whole-brain connectivity patterns (i.e., “connectivity states”) were identified over time,^14^ showing that CI patients with MS transitioned less fluidly between such states compared with cognitively preserved (CP) patients.^15^ Thus, less network adaptability might be particularly important for cognitive impairment in MS. Questions remain, including which specific adaptations are important for cognitive impairment? In addition, can structural network disturbances impede network adaptability by increasing energy costs?

Sensitivity to changes occurring on small temporal scales is needed to accurately characterize transitions between connectivity states. Nevertheless, connectivity dynamics have usually been captured using a sliding-window approach, which induces temporal blurring by computing correlations over windows of around a minute long.^16^ This can be ameliorated by temporally unwrapping correlation values and focusing on the resulting “edge time series,” which represent moment-to-moment cofluctuations of regional brain activity. This approach makes it possible to disentangle brief events of high-amplitude cofluctuations from nonevents.^16,17^ Disentangling these could be useful because both were related to cognitive performance and may provide unique insights.^18^

The structural network shapes brain functioning, so factors altering the structural network (e.g., damage or innate topology) can affect functional network dynamics too. The interaction between structure and function was related to cognitive impairment in MS,^19^ but an intuitive link explaining how the structural network could shape functional network dynamics has been missing. Network control theory can provide this, by modelling how complex functional patterns emerge from an underlying structural network. According to this framework, the wiring of the brain makes certain dynamic transitions occur naturally (i.e., natural trajectory), but other transitions require additional external input (i.e., control energy). It is important that it allows quantifying the control energy that is required for specific state transitions.^20^ Control energy has been likened to cognitive demand or mental load, so these measures quantify how effortful state transitions are. Recent work showed that physically disabled patients with MS required more control energy to transition between activity states,^21^ but it is unclear whether this can explain disrupted network dynamics in patients with cognitive impairment.

Therefore, this work aimed to increase our understanding of the functional underpinnings of cognitive impairment in MS. This is performed by characterizing framewise connectivity state transitions and computing the control energy required for these transitions. Based on abovementioned results pointing toward a hub overload, we hypothesized that MS patients with cognitive impairment would remain “stuck” in (i.e., transition away less from) states featuring relatively high connectivity of networks with many hub regions. This pattern was expected to be explained by the energetic costs of making the transitions.

## Methods

### Participants

Cross-sectional imaging from the Amsterdam MS cohort was analyzed, including patients with MS and healthy controls (HCs) based on the availability of functional and diffusion MRI data. Participants were recruited at the MS Center Amsterdam between 2008 and 2012. Functional network dynamics has been described previously for these participants,^12,22^ but not yet in combination with diffusion MRI data (for details, see eMethods 1). All patients were diagnosed with clinically definite MS according to the 2010 revised McDonald criteria.^3^ These patients were required to be relapse-free and without steroid treatment for 2 months before participation, and have no history of a psychiatric and/or neurologic disease besides MS. No other inclusion criteria related to treatment, disease duration, or other factors concerning overall disease severity were used. Age, sex, and the highest obtained level of education were acquired from all participants, and clinical data obtained from patients included symptom duration, disease phenotype, and treatment status. The Expanded Disability Status Scale (EDSS) was administered by a neurologist to characterize the level of physical disability. Fatigue was ascertained in a subset of patients (N = 167) with the checklist individual strength-20 revised.

### Standard Protocol Approvals, Registrations, and Patient Consents

Study approval was acquired from the institutional ethics review board of the Amsterdam UMC, location VUmc. All participants provided written informed consent before participation.

### Neuropsychological Assessment

An expanded Brief Repeatable Battery of Neuropsychological Tests was used on the same day as the MRI examination.^23^ Performance on individual tests was adjusted for age, sex, and education effects in the HCs and associated with a specific cognitive domain for descriptive purposes. Domains included executive functioning (concept shifting test), verbal memory (selective reminding test), information processing speed (symbol digit modalities test), verbal fluency (word list generation test), visuospatial memory (spatial recall test), working memory (memory comparison test), and attention (stroop color-word test). The paced auditory serial addition task was excluded because of extensive learning effects in our sample. The scores were transformed to *z*-scores based on the distribution of HCs. Performance on all domains was compared with HCs, resulting in 3 groups in MS: CI, mildly CI (MCI), and CP. Classification of CI patients was defined as scoring at least 2 SDs below HCs on 2 or more cognitive domains.^9^ Patients who were not defined as CI, but scored at least 1.5 SDs below HCs on 2 or more cognitive domains, were classified as MCI. All other patients were classified as CP.

### MRI Acquisitions

All scans were acquired using a 3T MRI scanner (GE Signa-HDxt, Milwaukee, WI) with an 8-channel phased-array head coil. The protocol included a 3D T1-weighted fast spoiled gradient echo sequence (repetition time [TR]/echo time [TE] = 7.8/3 milliseconds; inversion time = 450 milliseconds; flip angle = 12°; sagittal slice thickness = 1.0 mm; in-plane resolution = 0.9 × 0.9 mm), a 3D T2-weighted fluid-attenuated inversion recovery (FLAIR) sequence (TR/TE = 8,000/125 milliseconds; inversion time = 2,350 milliseconds; sagittal slice thickness = 1.2 mm; in-plane resolution = 1.0 × 1.0 mm), a resting-state fMRI echo planar imaging sequence (202 volumes; TR/TE = 2,200/35 milliseconds; flip angle = 80°; axial slice thickness = 3 mm, contiguous; in-plane resolution = 3.3 × 3.3 mm; eyes closed), and a diffusion tensor imaging sequence using 5 volumes without directional weighting (b = 0 s/mm^2^) and 30 with noncollinear diffusion gradients (b = 1,000 s/mm^2^, TR/TE = 13,000/91 milliseconds, flip angle = 90°, axial slice thickness = 2.4 mm, contiguous; in-plane resolution = 2 × 2 mm).

### Image Preprocessing

#### Lesion Detection and Filling

White matter lesions of patients with MS were automatically segmented on FLAIR images,^24^ and the resulting lesion masks were linearly registered to T1-space to perform lesion filling.^25^

#### Functional Preprocessing

The fMRI images of all 425 participants were preprocessed using the MELODIC pipeline (FSL 6),^26^ including the removal of the first 2 volumes, motion correction, brain extraction, and 4 mm Gaussian smoothing. Subsequently, ICA-AROMA (v0.4-beta)^27^ was used for automatic removal of residual motion artifacts. Then, regression of mean white matter and CSF signal was performed, followed by high-pass temporal filtering, boundary-based registration to T1-space, and coregistration and registration to standard space.

#### Diffusion Preprocessing

Complete diffusion MRI data were available for 420 participants. Preprocessing was performed using QSIPrep 0.14.3.^28^ This included denoising and correction for B1 field inhomogeneity, head motion, and eddy currents. Then, a deformation field was estimated using a registration-based fieldmap-less approach and used to calculate an unwarped b0 reference (warping constrained along the phase-encoding direction).^29^ The unwarped diffusion data were then registered to the T1-weighted volume with 2 mm isotropic voxels. Fiber response functions and orientation distributions (FODs) were produced with an unsupervised multitissue method and subsequent intensity normalization.^30^

### Structural Damage Indicators

#### Volumetric Measures

FreeSurfer 7.1.1 was performed on lesion-filled T1-weighted images and used to derive cortical gray matter volume.^31^ Deep gray matter volume was derived using FSL's FIRST. Both cortical and deep gray matter volumes were normalized for the estimated total intracranial volume by freesurfer. Lesion masks (see “Lesion detection and filling”) were used to determine white matter lesion volume.

#### White Matter Integrity

Fractional anisotropy (FA) was calculated for each voxel using DSI studio.^32^ FA maps were nonlinearly registered and projected onto an FA template skeleton, using the tract-based spatial statistics pipeline.^33^ The mean FA over the whole skeleton signified white matter integrity. This approach was chosen to minimize partial volume effects.

### Network Generation

#### Functional Networks

**Figure 1:**
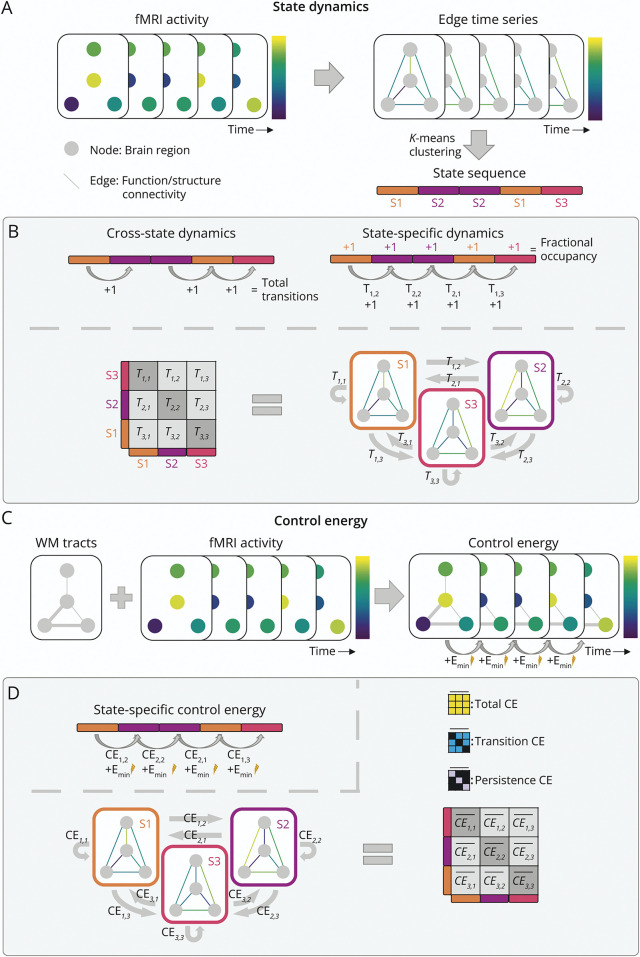
Illustration of the Quantification of State Dynamics and Control Energy (A) Functional MRI (fMRI) data were used to create edge time series, which reflected a functional network per time point. These networks were clustered using *k*-means clustering to define brain states (S1-S3 in this example; each represented by different colors). (B) Cross-state dynamics were quantified using the total number of transitions, whereas fractional occupancy (i.e., fraction of time spent in each state) was used as state-specific measure and the transition probability (T_i,j_: probability of transitioning from state *i* to state *j*, relative to the total transitions from *i*) as transition-specific measure. (C) Information on the number of streamlines of white matter (WM) tracts (based on diffusion MRI) was combined with fMRI data, to derive the minimum control energy (Emin) that is required to transition between successive frames. (D) The resulting Emin values were averaged over the same transitions (using the state sequence) to compute an energy transition matrix for each participant, so this matrix denotes the mean Emin required for each type of transition. The mean over the total matrix signified total control energy (CE), whereas the diagonal and off-diagonal reflected transition and persistence CE.

All 210 cortical regions from the Brainnetome atlas^34^ were combined with 14 deep gray matter (DGM) segmentations derived from FSL's FIRST and transformed to fMRI space. For visualization, all regions were assigned to 1 of 7 cortical resting-state subnetworks^35^ based on maximum overlap. All DGM regions were combined into 1 distinct network. Only voxels that represented gray matter were included, whereas a distorted resting-state fMRI signal was excluded from the atlas.^9^ Regions with less than 30% nondistorted voxels in more than 10% of participants were discarded (the bilateral orbitofrontal and nucleus accumbens). This resulted in 197 brain regions from which regional functional time series were extracted. We computed edge time series by transforming nodal time series to *z*-scores (using nodal means and SDs) and performing pointwise multiplication.^16,17^ Edge time series characterized a 197 × 197 functional network for each frame in the scan (Figure 1A).

#### Structural Networks

Tractography was performed using MRTrix3 using the normalized white matter FODs, by applying iFOD2 probabilistic tracking to generate 10 million streamlines.^36^ Anatomical constraints were provided by a hybrid surface/volume segmentation.^37^ Finally, streamline weights were calculated using SIFT2,^38^ and a 196 × 196 structural connectivity matrix was filled with the weighted number of streamlines connecting brain regions, using only regions in the functional network (excluding the cerebellum because of its structural complexity).

Registrations, response functions, and structural networks were visually inspected. No incoherent data were observed.

### State Dynamics

#### State Identification

Edge time series were concatenated across participants, and *k*-means clustering (MATLAB) was performed to derive 2–7 connectivity states,^14^ with 5 replicates and city block distance. The optimal number of states was derived using the elbow criterion, resulting in 4 states whose centroids (cluster median) represented robust coactivation patterns. The resulting state sequence assigns each frame to a connectivity state. The organization of these states was described by computing global mean connectivity, global efficiency, modularity, and the eigenvector centrality per resting-state network of the centroids, using the brain connectivity toolbox. The network with highest centrality was used to name the states.

#### State Dynamics Characterization

The total number of transitions across all states, as well as the average fractional occupancy (time spent in each state) and relative transition probabilities (probability of transitioning between and persisting within each individual state) were computed (Figure 1B).^14^ These transition probabilities are based on the temporal ordering of connectivity patterns, meaning that they are directional (i.e., [state 1 → state 2] ≠ [state 2 → state 1]). In line with previous research,^39^ relative transition probabilities from states that were not visited were considered missing values.

### Control Energy

The nctpy Python toolbox was used to determine control energy based on network control theory (see eMethods 2 for details). Minimum control energy (Emin) was calculated per brain region, reflecting the minimum external input that explains the observed change in brain activity (Figure 1C). The sum across brain regions defined overall required energy for a transition. Averaging Emin from the same state transitions using information from the state sequence, resulted in a 4 × 4 control energy transition matrix per participant (Figure 1D). Each transition in the transition matrix was transformed to a *z*-score based on the distribution of HCs for that transition. The average across the entire matrix determined the total control energy required across state transitions, representing overall energy inefficiency or energetic costs. The means of the diagonal values determined persistence control energy, denoting the costs of staying in the same state. The off-diagonal values were used for transition control energy, signifying the costs of transitioning to a different state. Some transitions were not observed in specific individuals, so the control energy for these transitions was regarded as missing values.

### Statistical Analyses

SPSS 28 was used for all statistical analyses. All group comparisons (unless differently specified) were adjusted for age, sex, and education. Education was based on the highest level attained and was binarized for analyses (higher vocational education or university yes/no). When the same analysis was performed across multiple states or transitions, multiple comparisons were taken into account using Bonferroni and corrected *p*-values were reported. An α-level of 0.05 was considered statistically significant. Normality of the dependent variables was inspected visually and using Kolmogorov-Smirnov tests.

Demographics and clinical variables were compared between the cognitive groups (HC, CP, MCI, and CI) using χ^2^ tests of independence for categorical variables and analysis of variances for numeric variables (no adjustment for covariates). For group comparisons of all imaging measures, linear mixed models were used when the data were normally distributed and Quade's nonparametric analysis of covariance if not. These tests allow finding ordered as well as nonordered effect across cognitive groups. Using this approach, differences in the total number of state transitions and the fractional occupancy of each specific state were compared between cognitive groups. Transition probabilities were investigated for states that showed differences between groups. Total control energy as well as transition and persistence control energy were compared between the cognitive groups. Then, the difference in control energy required to transition between specific states was evaluated. Finally, the connection between transition probability and control energy was investigated in relation to measures of structural damage and clinical indicators of MS using partial correlations.

### Data Availability

Anonymized data, not published in the article, will be shared on reasonable request from a qualified investigator. Code is available on GitHub.^40^

## Results

### Demographics and Clinical Characteristics

Complete fMRI and neuropsychological assessments were available for 330 patients (mean age of 48 ± 11 years; 68% female) and 95 HCs (mean age of 46 ± 10 years, 58% female). Across all patients, 179 (54%) were classified as CP (131 women, mean age: 46 ± 10 years), 65 (20%) as MCI (42 women, mean age: 49 ± 12 years), and 86 (26%) as CI (51 women, mean age: 51 ± 11 years). Cognitive groups differed on age, sex, and educational level (Table 1). Finally, CI patients showed most gray matter atrophy and lesion volume.

**Table 1:** Demographic, Clinical, and Brain Volumetric Sample Characteristics

	HC (N = 95)	MS			Test statistic	*p* Value
		CP (N = 179)	MCI (N = 65)	CI (N = 86)		
Demographics						
Male, n	40 (42.1%)	48 (26.8%)	23 (35.4%)	35 (40.7%)	χ^2^ = 8.607	0.035^a^
Age, y	45.70 ± 10.35^CI^	46.16 ± 10.35^CI^	49.19 ± 12.15	51.21 ± 10.66^HC,CP^	*F* = 5.819	<0.001^a^
Level of education^b^	6 (3)^MCI,CI^	6 (2)^MCI,CI^	4 (3)^HC,CP^	4 (3)^HC,CP^	*F* = 7.035	<0.001^a^
Handedness, left/right/missing	0/0/95	24/153/2	3/61/1	10/76/0	χ^2^ = 3.698	0.157
Disease characteristics						
Symptom duration	—	13.49 ± 7.83^CI^	14.15 ± 8.15	17.17 ± 9.30^CP^	*F* = 5.799	0.003^a^
Disease phenotype, RRMS/SPMS/PPMS	—	147^CI^/20^CI^/12^MCI^	47/6^CI^/12^CP^	49^CP^/25^CP,MCI^/12	χ^2^ = 26.106	0.001^a^
Treatment, yes, n	—	63 (35.2%)	26 (40.0%)	57 (33.7%)	χ^2^ = 0.689	0.709
IFB/COP/NA/other	—	37/6/16/4	18/6/1/1	18/4/6/1	χ^2^ = 7.666	0.264
EDSS^b^	—	3 (2)^CI^	3 (1.5)^CI^	4 (2.75)^CP,MCI^	*F* = 25.360	<0.001^a^
Brain volume						
Cortical GM, BPF (%)	32.28 ± 1.52^CI^	31.99 ± 1.77^CI^	31.69 ± 1.99^CI^	30.59 ± 1.71^All^	*F* = 16.823	<0.001^a^
Deep GM, BPF (%)	3.01 ± 0.21^All^	2.78 ± 0.29^All^	2.65 ± 0.33^All^	2.40 ± 0.41^All^	*F* = 61.422	<0.001^a^
Lesion volume, mL	—	10.28 (8.63)^All^	14.05 (11.02)^All^	22.09 (17.00)^All^	*F* = 21.126	<0.001^a^

Abbreviations: BPF = brain parenchymal fraction; CI = cognitive impairment; cop = copaxone; CP = cognitively preserved; EDSS = Expanded Disability Status Scale; GM = gray matter; HC = healthy control; IFB = interferon β; MCI = mild cognitive impairment; MS = multiple sclerosis; NA = natalizumab; PPMS = primary progressive MS; RRMS = relapsing-remitting MS; SPMS = secondary progressive MS.

All values represent means and SDs for the continuous variables but signify medians and the interquartile range (^b^) or frequencies for categorical variables. Sample characteristics were compared between all groups. The level of education was based on the highest level of education attained. Brain lesion volume was transformed to milliliters for readability. Post hoc pairwise comparisons were Bonferroni corrected (^All^ = significantly different from all other groups, ^HC^ = significantly different from HC, ^CP^ = significantly different from CP, ^MCI^ = significantly different from MCI, ^CI^ = significantly different from CI).

a *p* < 0.05.

### State Organization

**Figure 2:**
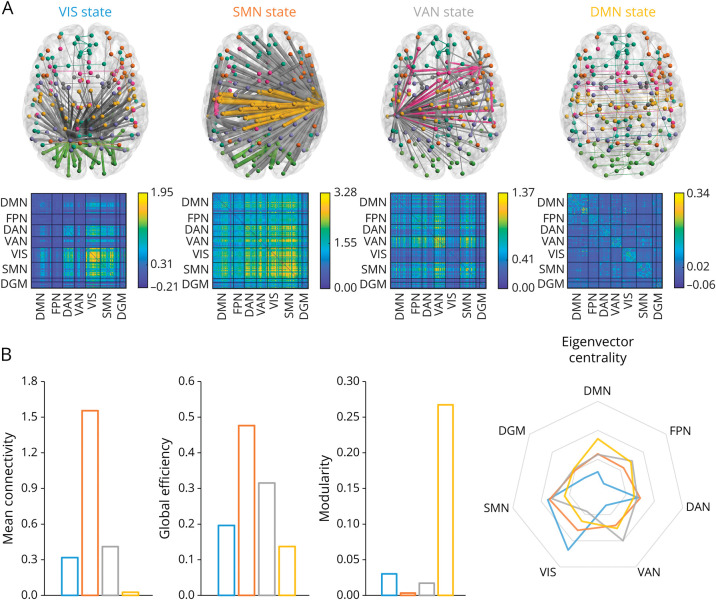
Brain State Organization Four states were identified using *k*-means clustering. (A) The backbone of the network of the state (minimum spanning tree) is depicted, with the thickness indicating connection strength and the colored edges indicating within-network connections and gray edges between network connections. The corresponding connectivity matrices are depicted below. (B) Global connectivity strength, global efficiency, global modularity, and the mean eigenvector centrality per resting-state network are portrayed per state centroid to help illustrate how states differed from each other. DAN = dorsal attention network; DGM = deep gray matter; DMN = default-mode network; FPN = frontoparietal network; SMN = sensorimotor network; VAN = ventral attention network; VIS = visual network.

Four connectivity states were identified (Figure 2A). The first state was moderately connected with relatively high centrality of the sensorimotor network (SMN) but highest in the VIS, because of which it was described as the VIS state (Figure 2B). The second state was strongly connected overall, most notably in the SMN, and thus termed the SMN state. The third state showed moderate connectivity strength, with highest centrality in the ventral attention network (VAN) and therefore called the VAN state. The fourth state was highly modular with overall weak connectivity and highest centrality in the DMN; thus, it was named the DMN state. These state descriptions are merely qualitative in nature and included to facilitate readability.

### State Dynamics

#### Total Transitions

**Figure 3:**
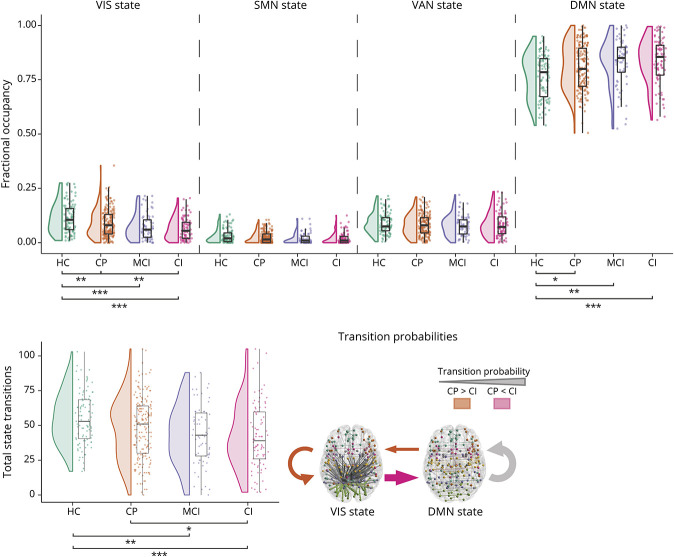
Brain State Dynamics Across Cognitive Groups In CI patients, fewer transitions between brain states were observed compared with CP and HCs. These patients spend less time in the VIS state and more time in the DMN state. CI patients show a lower probability to move from the DMN state to the VIS state and stay there relative to preserved patients, whereas the transition from the VIS state to the DMN state was more likely. The thickness of the arrows on the bottom right indicates the relative probability of that transition occurring on average in HCs. CI = cognitively impaired; CP = cognitively preserved; DMN = default-mode network; HC = healthy control; MCI = mildly impaired; VIS = visual network. *p < 0.05, **p < 0.01, ***p < 0.001.

The transition frequency (*F*(3,418) = 4.92, *p* = 0.002) was lower in CI compared with CP patients and HCs (Figure 3 and Table 2). MCI patients also transitioned less frequently compared with HCs. Thus, dynamics became less fluid in impaired patients.

**Table 2:** State Dynamics and Control Energy Across Cognitive Groups

	Mean (±SD)				Main: Group		CI vs CP	
	HC (N = 95)	CP (N = 179)	MCI (N = 65)	CI (N = 86)	*F*	*p* Value	β (95% CI)	*p* Value
State dynamics								
Total transitions	54.9 (18.7)^MCI,CI^	48.0 (22.3)	43.3 (21.6)^HC^	42.1 (23.4)^HC,CP^	4.92	0.002^a^	−5.78 (−11.23, −0.32)	0.038^a^
Fractional occupancy								
VIS state	0.11 (0.07)^MCI,CI^	0.08 (0.06)^HC^	0.07 (0.06)^HC^	0.06 (0.06)^HC,CP^	9.38	<0.001^a^	−0.02 (−0.04, −0.01)	0.004^a^
SMN state	0.03 (0.03)	0.02 (0.03)	0.02 (0.03)	0.02 (0.03)	2.53	0.226		
VAN state	0.09 (0.05)	0.08 (0.05)	0.08 (0.05)	0.08 (0.06)	0.25	1.000		
DMN state	0.77 (0.10)^All^	0.81 (0.10)^HC^	0.83 (0.11)^HC^	0.83 (0.10)^HC^	5.55	0.004^a^	0.03 (0.00, 0.05)	0.053
Transition probability								
VIS state persist (NA = 14)	0.29 (0.18)^MCI,CI^	0.27 (0.18)^CI^	0.22 (0.18)^HC^	0.20 (0.20)^HC,CP^	4.34	0.020^a^	−0.06 (−0.11, −0.02)	0.007^a^
VIS state → DMN state (NA = 14)	0.57 (0.20)	0.62 (0.21)	0.63 (0.25)	0.70 (0.24)^HC,CP^	4.05	0.030^a^	0.08 (0.02, 0.14)	0.005^a^
DMN state persist	0.84 (0.07)^All^	0.86 (0.07)^HC^	0.88 (0.07)^HC^	0.88 (0.08)^HC^	4.62	0.014^a^	0.02 (0.00, 0.04)	0.062
DMN state → VIS state	0.08 (0.05)^CI^	0.07 (0.05)^CI^	0.05 (0.04)	0.05 (0.04)^HC,CP^	7.99	<0.001^a^	−0.02 (−0.03, −0.01)	0.004^a^

	HC (N = 95)	CP (N = 176)	MCI (N = 63)	CI (N = 86)	*F*	*p* Value	β (95% CI)	*p* Value
Control energy								
Total control energy	0.09 (0.57)^MCI,CI^	0.38 (0.73)^MCI,CI^	0.66 (1.23)^HC,CP^	0.57 (0.88)^HC,CP^	6.56	<0.001^a^	0.25 (0.04, 0.46)	0.018^a^
Persistence energy	0.10 (0.73)	0.35 (0.92)	0.64 (1.03)	0.53 (0.94)	2.99	0.061		
Transition energy (NA = 2)	0.08 (0.58)^MCI,CI^	0.24 (0.76)^MCI,CI^	0.62 (1.35)^HC,CP^	0.58 (1.00)^HC,CP^	6.19	<0.001^a^	0.32 (0.09, 0.54)	0.007^a^
Transition energy								
VIS state persist (NA = 93^b^)	0.00 (1.00)	0.05 (1.07)	0.28 (1.84)	0.01 (1.21)	0.53	1.000		
VIS state → DMN state (NA = 17)	0.00 (1.00)	0.12 (1.06)	0.40 (1.53)	0.47 (1.38)	2.24	0.332		
DMN state persist	0.00 (1.00)	0.38 (1.07)	0.66 (1.18)	0.47 (1.03)	3.30	0.084		
DMN state → VIS state (NA = 15)	0.00 (1.00)^MCI,CI^	0.11 (0.93)^MCI,CI^	0.64 (1.75)^HC,CP^	0.51 (1.64)^HC,CP^	3.87	0.038^a^	0.34 (0.00, 0.67)	0.049^a^

The total frequency of transitions was lower in cognitively impaired (CI) patients compared with preserved (CP) patients and healthy controls (HCs). CI patients spent relatively less time in the VIS state and more in the DMN state, with transition probabilities highlighting a general pattern of more transitions toward the DMN state and away from the VIS state. The total control energy and particularly the control energy associated with transitions were elevated in CI and mildly cognitively impaired (MCI) patients compared with CP patients and HCs. This was most notable for the transition from the DMN state to the VIS state. If a participant did not access a particular state, this occasionally resulted in missing values (NA; i.e., not available).

The reported *p* values for the main group effects were corrected for multiple comparisons using Bonferroni, and pairwise comparisons are reported if the corrected *p* < 0.05 (^All^ = different from all other groups, ^HC^ = different from HC, ^CP^ = different from CP, ^MCI^ = different from MCI, ^CI^ = different from CI).

a *p* < 0.05.

b The proportion of missing values was higher for MCI and CI patients compared with controls and CP patients.

#### Fractional Occupancy

VIS state fractional occupancy (*F*(3,418) = 9.38, *p* < 0.001) was significantly lower in CI compared with CP patients and HCs. Both MCI and CP patients also showed a lower VIS state fractional occupancy compared with HCs. For the SMN state (*F*(3,418) = 2.53, *p* = 0.226) and the VAN state (*F*(3,418) = 0.25, *p* = 1.000), the same directionality was observed, but no significant group differences were found. The DMN state fractional occupancy (*F*(3,418) = 5.55, *p* = 0.004) showed the opposite effect, being higher in CI patients compared with HCs. Although it was not significantly elevated in CI compared with CP patients, all patients showed higher DMN state occupancy compared with HCs. These findings indicate that the time spent in the VIS state and DMN state was altered in MS, with impaired patients spending less time in the VIS state.

#### Transition Probabilities

Based on the results above, the persistence and transition probabilities of the VIS state and DMN state were explored further. For VIS state persistence (*F*(3,404) = 4.34, *p* = 0.020), lower probabilities were observed in CI compared with CP patients and HCs. MCI also showed lower VIS state persistence probability compared with HCs. For DMN state persistence (*F*(3,418) = 4.62, *p* = 0.014), differences were not observed between patients and only a heightened probability was observed compared with HCs across all patient groups. Regarding the VIS → DMN transition (*F*(3,404) = 4.05, *p* = 0.030), CI showed higher probabilities compared with CP patients and HCs. The inverse was true for the DMN → VIS transition (*F*(3,418) = 7.99, *p* < 0.001) because CI showed a lower probability compared with CP.

### Control Energy

#### Total Control Energy

**Figure 4:**
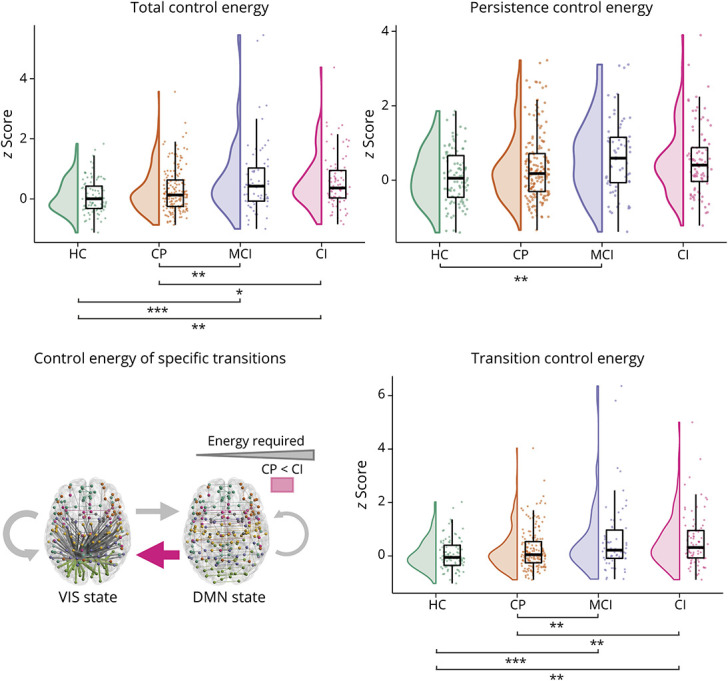
Control Energy of Brain State Transitions Across Cognitive Groups In CI patients, transitions between and within brain states were more energetically costly compared with CP and HCs. In particular, more control energy was required for transition between states, not for persisting in the same state. The transition from the DMN state to the VIS state was particularly more costly in CI compared with CP patients. The thickness of the arrows on the bottom left indicates how much control energy is required on average in HCs for that transition. CI = cognitively impaired; CP = cognitively preserved; DMN = default-mode network; HC = healthy control; MCI = mildly impaired; VIS = visual network. *p < 0.05, **p < 0.01, ***p < 0.001.

Of 330 patients, 5 had incomplete diffusion MRI data (3 CP and 2 MCI) and were excluded from these analyses. Total control energy (*F*(3,413) = 6.56, *p* < 0.001) was increased in MCI and CI compared with CP patients and HCs (Figure 4 and Table 2).

#### Persistence and Transition Control Energy

Persistence control energy was not different between groups (*F*(3,413) = 2.99, *p* = 0.061); thus, staying in the same state was not more energetically costly in CI. Transition control energy (*F*(3,411) = 6.19, *p* < 0.001) was increased in MCI and CI compared with CP patients and HCs. This indicated that CI featured more energetically costly transitions between states. Based on these findings, we used transition and not total control energy in the correlations with disease severity.

#### Energy of Specific State Transitions

Similarly to transition probability, Emin of VIS state and DMN state transitions were compared between groups. Only DMN → VIS transition energy showed significant differences between groups (*F*(3,398) = 3.87, *p* = 0.038), with CI and MCI patients both showing increased energy compared with CP patients and HCs. VIS → DMN transition energy (*F*(3,396) = 2.24, *p* = 0.332), as well as VIS state persistence energy (*F*(3,320) = 0.53, *p* = 1.000) or DMN state (*F*(3, 413) = 3.30, *p* = 0.084) did not differ between groups.

Exploratory analyses showed a stepwise worsening of structural network disruption in higher-order networks and the DGM (eMethods 3).

### Validation Analyses

For most analyses showing group differences, age and sex were significant covariates and education was not. For VIS → DMN transition probability, only age was a significant covariate. Differences between CI and CP patients were largely retained when comparing only right-handed individuals, with only a trend observed for DMN → VIS transition energy (possibly due to power; eMethods 4).

### Correlations With Disease Severity

#### Structural Damage

Reduced white matter integrity in patients with MS related to less frequent state transitions (*r*(320) = 0.16, *p* = 0.013), an increased DMN state persistence (*r*(320) = −0.16, *p* = 0.017), more transition control energy (*r*(318) = −0.15, *p* = 0.023), and more VIS → DMN transition energy (*r*(303) = −0.17, *p* = 0.010; Table 3). No relationship with gray matter volume was observed (validated using cortical thickness; eMethods 5).

**Table 3:** Correlational Analyses

	Total transitions (N = 330)		Transition control energy (N = 323)		VIS state persist				VIS → DMN				DMN state persist				DMN → VIS			
					Probability (N = 316)		Control energy (N = 243)		Probability (N = 316)		Control energy (N = 308)		Probability (N = 330)		Control energy (N = 325)		Probability (N = 330)		Control energy (N = 310)	
	*r*	*p* Value	*r*	*p* Value	*r*	*p* Value	*r*	*p* Value	*r*	*p* Value	*r*	*p* Value	*r*	*p* Value	*r*	*p* Value	*r*	*p* Value	*r*	*p* Value
Structural damage																				
Cortical GM volume	0.13	0.085	−0.08	0.689	0.04	1.000	−0.12	0.268	−0.03	1.000	−0.08	0.646	−0.14	0.057	−0.05	1.000	0.02	1.000	−0.08	0.660
Deep GM volume	0.07	0.833	−0.02	1.000	0.05	1.000	−0.05	1.000	−0.10	0.344	−0.02	1.000	−0.06	1.000	0.04	1.000	0.02	1.000	−0.01	1.000
Lesion volume	−0.07	0.925	0.00	1.000	−0.02	1.000	0.03	1.000	0.01	1.000	0.03	1.000	0.06	0.967	−0.04	1.000	−0.06	0.993	0.04	1.000
WM integrity	0.16	0.013^a^	−0.15	0.023^a^	0.13	0.092	−0.04	1.000	−0.10	0.295	−0.17	0.010^a^	−0.16	0.017^a^	−0.06	1.000	0.09	0.464	−0.08	0.580
Clinical outcomes																				
EDSS	−0.16	0.010^a^	0.09	0.184	−0.12	0.054	−0.01	1.000	0.15	0.013^a^	0.16	0.008^a^	0.15	0.013^a^	0.08	0.301	−0.13	0.046^a^	0.13	0.051
Fatigue	−0.10	0.412	0.08	0.659	−0.11	0.352	0.14	0.268	0.16	0.084	0.21	0.020	0.11	0.332	0.03	1.000	−0.17	0.065	0.08	0.697
Cognitive domains																				
Executive functioning	0.08	1.000	−0.12	0.221	0.10	0.484	0.09	1.000	−0.13	0.146	−0.16	0.041	−0.06	1.000	0.03	1.000	0.08	1.000	−0.14	0.130
Verbal memory	0.09	0.693	−0.04	1.000	0.04	1.000	−0.11	0.599	−0.07	1.000	0.03	1.000	−0.08	1.000	−0.04	1.000	0.07	1.000	0.02	1.000
Processing speed	0.17	0.014^a^	−0.22	0.001^a^	0.16	0.040^a^	−0.08	1.000	−0.14	0.093	−0.15	0.077	−0.15	0.045^a^	−0.09	0.799	0.11	0.279	−0.16	0.047^a^
Verbal fluency	0.09	0.765	−0.19	0.004^a^	0.08	1.000	−0.12	0.382	−0.09	0.806	−0.13	0.181	−0.08	1.000	−0.01	1.000	0.08	1.000	−0.15	0.055
Visuospatial memory	0.06	1.000	−0.06	1.000	0.13	0.137	0.03	1.000	−0.07	1.000	−0.04	1.000	−0.05	1.000	−0.08	1.000	0.06	1.000	−0.13	0.155
Working memory	0.06	1.000	−0.10	0.508	0.11	0.455	0.13	0.352	−0.09	0.839	−0.01	1.000	−0.05	1.000	−0.07	1.000	0.04	1.000	−0.05	1.000
Attention	0.11	0.321	−0.07	1.000	0.17	0.020^a^	−0.09	1.000	−0.20	0.004^a^	0.00	1.000	−0.10	0.627	−0.08	1.000	0.11	0.310	−0.03	1.000

Abbreviations: BPF = brain parenchymal fraction; EDSS = Expanded Disability Status Scale; GM = gray matter; WM = white matter.

These correlational analyses were performed on data from MS patients (N = 330), with missing values for WM integrity (5), fatigue (163), executive functioning (12), verbal memory (2), IPS (2), verbal fluency (1), working memory (12), and attention (12). All correlations were adjusted for age, sex, and level of education. The *p* values were corrected for performing multiple comparisons using Bonferroni.

a *p* < 0.05.

#### Clinical Outcomes

Worse EDSS related to fewer transitions (*r*(325) = −0.16, *p* = 0.010), a higher VIS → DMN transition probability (*r*(303) = 0.15, *p* = 0.013), increased DMN state persistence probability (*r*(325) = 0.15, *p* = 0.013), and DMN → VIS transition probability (*r*(325) = −0.13, *p* = 0.046). Worse EDSS also related to more VIS → DMN transition energy (*r*(311) = 0.16, *p* = 0.008).

#### Cognitive Domains

Poorer information processing speed related to fewer transitions (*r*(323) = −0.17, *p* = 0.014). Worse information processing speed (*r*(316) = −0.22, *p* = 0.001) and verbal fluency (*r*(317) = −0.19, *p* = 0.004) associated with elevated transition energy in MS. Lower information processing speed (*r*(309) = 0.16, *p* = 0.040) and attention (*r*(299) = 0.17, *p* = 0.020) related to less VIS state persistence probability. Poorer attention related to more VIS → DMN transition probability (*r*(299) = −0.20, *p* = 0.004). Lower processing speed related to higher DMN state persistence probability (*r*(323) = −0.15, *p* = 0.045). Finally, poorer processing speed related to more DMN → VIS transition energy (*r*(303) = −0.16, *p* = 0.047).

## Discussion

This study showed that dynamic network changes required for normal cognitive processing require more effort in CI people with MS because transitions between connectivity states required more control energy. This suggests that state transitions became more effortful and may explain why transitions happen less frequently in CI patients with MS. The results showed that impaired patients spent more time in the DMN state and less in the VIS state, with the probability of transitioning to and from the VIS state being altered in CI patients. Transitions that happened more frequently did not become less energetically costly, but patients who required more control energy generally transitioned toward the DMN state and away from the VIS state.

CI and MCI used more control energy for transitions between connectivity states. This provides a mechanistic understanding of reduced brain dynamics in MS patients with cognitive impairment, suggesting that state transitions became more cognitively and metabolically demanding,^20^ which may prevent transitions from occurring. An alternative possibility is that transitions occurring less frequently become more energetically costly. However, MCI patients only showed differences from CP for control energy and not state dynamics, suggesting more costly dynamics may precede abnormal state transitions over the disease course. Several possible explanations exist for more energetically costly network dynamics in MS. First, demyelination-related conduction delays in MS can affect efficient information transfer,^41^ which matches our observed link with white matter integrity. We detected no relationship with lesion volume or atrophy in MS. This suggests that demyelination severity could affect these measures more strongly than changes in brain morphometry or diffuse demyelination, which can be further studied using modelling work with individualized estimations of conduction velocities.^42^ Second, more energetically costly transitions may be linked to an excitation-inhibition imbalance^43^ because an adequate balance is critical for efficient neural encoding^44^ and cognition in MS.^45^ Third, structural damage in MS might impair the efficient wiring and make transitions more energetically costly.^46^

The VIS state was especially important for cognition because CI-MS was less likely to stay in this state and more likely to move to the DMN state than CP patients. Conversely, when in the DMN state, CI patients moved to the VIS state less. Thus, CI patients did not only get “stuck” in states featuring relatively high connectivity of hub networks (e.g., the DMN)^7,9,12^ but also returned to them more often. Moreover, the DMN state was weakly connected, which may not align with an overload of hubs. This weakly connected state was the most abundant across participants, which was further elevated in CI patients. Lower connectivity strength is arguably less metabolically demanding,^16^ so residing in this state could be a compensatory mechanism. Periods of low connectivity may be uniquely relevant for cognition^18^ but were likely underrepresented in static or windowed approaches,^16^ emphasizing the utility of framewise approaches. Periods of more integrative connectivity (the VIS state) were observed less in CI patients, whereas integrated processing is important for complex cognitive tasks^47^ which is often impaired in MS.^1^ Alternative ways to integrate information across the network may be important for CI patients because heightened dynamic connectivity was observed when quantifying dynamics using an approach that is more sensitive to nonhub integration.^13^ Hampered transitions from the DMN state to the VIS state might reflect disrupted switching between internally and externally oriented processing, as previously proposed.^12^ No differences in state dynamics were observed for the SMN state and VAN state. Future work should investigate whether these states are less important for cognitive performance or merely not sufficiently engaged at rest.

The total transition frequency may be a broad indicator of disease severity because we also observed that fewer transitions related to more physical disability. This aligns with prior research on connectivity states in MS.^15,48^ The same might be true for the increase in control energy for CI patients because recent work showed that MS patients with physical disability required more control energy.^21^ By contrast, we did not observe a relationship between physical disability and transition control energy, possibly because our cohort had longer disease durations where disability mechanisms might be different. Of interest, transition control energy was particularly relevant for information processing speed and verbal fluency, suggesting that it might be related to shared cognitive processes, such as cognitive flexibility. Altered network dynamics could theoretically affect fatigue in MS, given the observed link between fatigue and energy metabolism.^49^ We did not observe such a relationship, so dedicated studies now need to test its importance for specific types of fatigue.

Disturbed network dynamics have been reported for several other brain disorders, so our framework provides a broadly applicable new perspective to link brain function to structural network organization. Although control energy should not be directly equated to metabolic energy, previous work did show a relationship between control energy and glucose metabolism.^46^ Parameter choice is still a topic of debate, however, which is why we used data-driven optimization of the control horizon.^21^ Despite these challenges, the current framework offers an exciting opportunity and generalizable approach to study and develop personalized treatment of cognitive impairment in MS.^50,51^ Furthermore, although fine-grained temporal scales can increase noisiness of windowed connectivity, the current approach uses information (e.g., variance) from the entire scan and is not affected by noisy estimations in the same way.^17^ Higher b-values, more phase-encoding directions, and isotropic acquisitions were recommended for structural network generation, warranting future work to use advanced diffusion protocols that might yield more sensitive markers of cognitive dysfunction in MS. The chance of sojourning in the same state was particularly low for event states (i.e., brief high-amplitude cofluctuations), so other modalities that acquire data with a better temporal resolution (e.g., electroencephalography/magnetoencephalography) or that explicitly take the temporal sequence into account when defining states may provide more detailed insights. Finally, explicit stimuli (e.g., tasks or movies) would be required to understand the cognitive processes underlying specific states.^52^

This study showed that transitions between connectivity states cost more energy in MS patients with CI compared with CP patients and controls. Heightened energetic costs might limit the transitions between states and, in turn, negatively affect cognition. The transitions between a DMN state and VIS state seem to be particularly relevant for cognition in these patients. These findings provide new insights into the possible biological underpinnings of disturbed brain dynamics in CI patients with MS. Future work should now focus on investigating these patterns across different disease stages.

## Appendix Authors

**Table:**

Name	Location	Contribution
Tommy A.A. Broeders, MSc	MS Center Amsterdam, Anatomy & Neurosciences, Vrije Universiteit Amsterdam, Amsterdam Neuroscience, Amsterdam UMC location VUmc, the Netherlands	Drafting/revision of the manuscript for content, including medical writing for content; study concept or design; analysis or interpretation of data
Maureen van Dam, MSc	MS Center Amsterdam, Anatomy & Neurosciences, Vrije Universiteit Amsterdam, Amsterdam Neuroscience, Amsterdam UMC location VUmc, the Netherlands	Drafting/revision of the manuscript for content, including medical writing for content
Giuseppe Pontillo, MD, PhD	MS Center Amsterdam, Radiology and Nuclear Medicine, Vrije Universiteit Amsterdam, Amsterdam Neuroscience, Amsterdam UMC location VUmc, the Netherlands; Queen Square Institute of Neurology and Centre for Medical Image Computing, University College London, United Kingdom; Departments of Advanced Biomedical Sciences and Electrical Engineering and Information Technology, University of Naples “Federico II,” Italy	Drafting/revision of the manuscript for content, including medical writing for content
Vasco Rauh, MSc	MS Center Amsterdam, Anatomy & Neurosciences, Vrije Universiteit Amsterdam, Amsterdam Neuroscience, Amsterdam UMC location VUmc, the Netherlands	Drafting/revision of the manuscript for content, including medical writing for content; study concept or design
Linda Douw, PhD	MS Center Amsterdam, Anatomy & Neurosciences, Vrije Universiteit Amsterdam, Amsterdam Neuroscience, Amsterdam UMC location VUmc, the Netherlands	Drafting/revision of the manuscript for content, including medical writing for content
Ysbrand D. van der Werf, PhD	MS Center Amsterdam, Anatomy & Neurosciences, Vrije Universiteit Amsterdam, Amsterdam Neuroscience, Amsterdam UMC location VUmc, the Netherlands	Drafting/revision of the manuscript for content, including medical writing for content
Joep Killestein, MD, PhD	MS Center Amsterdam, Neurology, Vrije Universiteit Amsterdam, Amsterdam Neuroscience, Amsterdam UMC location VUmc, the Netherlands	Drafting/revision of the manuscript for content, including medical writing for content
Frederik Barkhof, MD, PhD, FRCR	MS Center Amsterdam, Radiology and Nuclear Medicine, Vrije Universiteit Amsterdam, Amsterdam Neuroscience, Amsterdam UMC location VUmc, the Netherlands; Queen Square Institute of Neurology and Centre for Medical Image Computing, University College London, United Kingdom	Drafting/revision of the manuscript for content, including medical writing for content
Christiaan H. Vinkers, MD, PhD	MS Center Amsterdam, Anatomy & Neurosciences, and MS Center Amsterdam, Psychiatry, Vrije Universiteit Amsterdam, Amsterdam Neuroscience, Amsterdam UMC location VUmc; Amsterdam Public Health, Mental Health Program; GGZ inGeest Mental Health Care, Amsterdam, the Netherlands	Drafting/revision of the manuscript for content, including medical writing for content
Menno M. Schoonheim, PhD	MS Center Amsterdam, Anatomy & Neurosciences, Vrije Universiteit Amsterdam, Amsterdam Neuroscience, Amsterdam UMC location VUmc, the Netherlands	Drafting/revision of the manuscript for content, including medical writing for content; study concept or design; analysis or interpretation of data

## Glossary

CI: cognitively impaired
CP: cognitively preserved
DGM: deep gray matter
DMN: default-mode network
EDSS: Expanded Disability Status Scale
Emin: minimum control energy
FA: fractional anisotropy
FLAIR: fluid-attenuated inversion recovery
FOD: functions and orientation distribution
FPN: frontoparietal network
HC: healthy control
MCI: mildly CI
MS: multiple sclerosis
SMN: sensorimotor network
TE: echo time
TR: repetition time
VAN: ventral attention network
VIS: visual network

## References

[1] Benedict RHB, Amato MP, DeLuca J, Geurts JJG. Cognitive impairment in multiple sclerosis: clinical management, MRI, and therapeutic avenues. Lancet Neurol. 2020;19(10):860-871. doi:10.1016/S1474-4422(20)30277-5 32949546 PMC10011205

[2] Lassmann H. Multiple sclerosis pathology. Csh Perspect Med. 2018;8(3):a028936. doi:10.1101/cshperspect.a028936 PMC5830904 29358320

[3] Polman CH, Reingold SC, Banwell B, et al. Diagnostic criteria for multiple sclerosis: 2010 revisions to the McDonald criteria. Ann Neurol. 2011;69(2):292-302. doi:10.1002/ana.22366 21387374 PMC3084507

[4] Barkhof F. The clinico-radiological paradox in multiple sclerosis revisited. Curr Opin Neurol. 2002;15(3):239-245. doi:10.1097/00019052-200206000-00003 12045719

[5] Meijer KA, Steenwijk MD, Douw L, Schoonheim MM, Geurts JJG. Long-range connections are more severely damaged and relevant for cognition in multiple sclerosis. Brain. 2020;143(1):150-160. doi:10.1093/brain/awz355 31730165 PMC6938033

[6] Bassett DS, Sporns O. Network neuroscience. Nat Neurosci. 2017;20(3):353-364. doi:10.1038/nn.4502 28230844 PMC5485642

[7] Schoonheim MM, Broeders TAA, Geurts JJG. The network collapse in multiple sclerosis: an overview of novel concepts to address disease dynamics. Neuroimage Clin. 2022;35:103108. doi:10.1016/j.nicl.2022.103108 35917719 PMC9421449

[8] Schoonheim MM, Meijer KA, Geurts JJ. Network collapse and cognitive impairment in multiple sclerosis. Front Neurol. 2015;6:82. doi:10.3389/fneur.2015.00082 25926813 PMC4396388

[9] Eijlers AJ, Meijer KA, Wassenaar TM, et al. Increased default-mode network centrality in cognitively impaired multiple sclerosis patients. Neurology. 2017;88(10):952-960. doi:10.1212/WNL.0000000000003689 28179464

[10] Preti MG, Bolton TA, Van De Ville D. The dynamic functional connectome: state-of-the-art and perspectives. Neuroimage. 2017;160:41-54. doi:10.1016/j.neuroimage.2016.12.061 28034766

[11] Betzel RF, Fukushima M, He Y, Zuo XN, Sporns O. Dynamic fluctuations coincide with periods of high and low modularity in resting-state functional brain networks. Neuroimage. 2016;127:287-297. doi:10.1016/j.neuroimage.2015.12.001 26687667 PMC4755785

[12] Eijlers AJC, Wink AM, Meijer KA, Douw L, Geurts JJG, Schoonheim MM. Reduced network dynamics on functional MRI signals cognitive impairment in multiple sclerosis. Radiology. 2019;292(2):449-457. doi:10.1148/radiol.2019182623 31237498

[13] Broeders TAA, Douw L, Eijlers AJC, et al. A more unstable resting-state functional network in cognitively declining multiple sclerosis. Brain Commun. 2022;4(2):fcac095. doi:10.1093/braincomms/fcac095 35620116 PMC9128379

[14] Allen EA, Damaraju E, Plis SM, Erhardt EB, Eichele T, Calhoun VD. Tracking whole-brain connectivity dynamics in the resting state. Cereb Cortex. 2014;24(3):663-676. doi:10.1093/cercor/bhs352 23146964 PMC3920766

[15] d'Ambrosio A, Valsasina P, Gallo A, et al. Reduced dynamics of functional connectivity and cognitive impairment in multiple sclerosis. Mult Scler. 2020;26(4):476-488. doi:10.1177/1352458519837707 30887862

[16] Zamani Esfahlani F, Jo Y, Faskowitz J, et al. High-amplitude cofluctuations in cortical activity drive functional connectivity. Proc Natl Acad Sci USA. 2020;117(45):28393-28401. doi:10.1073/pnas.2005531117 33093200 PMC7668041

[17] Sporns O, Faskowitz J, Teixeira AS, Cutts SA, Betzel RF. Dynamic expression of brain functional systems disclosed by fine-scale analysis of edge time series. Netw Neurosci. 2021;5(2):405-433. doi:10.1162/netn_a_00182 34189371 PMC8233118

[18] Iraji A, Faghiri A, Fu Z, et al. Moving beyond the ‘CAP’ of the Iceberg: intrinsic connectivity networks in fMRI are continuously engaging and overlapping. Neuroimage. 2022;251:119013. doi:10.1016/j.neuroimage.2022.119013 35189361 PMC9107614

[19] Kulik SD, Nauta IM, Tewarie P, et al. Structure-function coupling as a correlate and potential biomarker of cognitive impairment in multiple sclerosis. Netw Neurosci. 2022;6(2):339-356. doi:10.1162/netn_a_00226 35733434 PMC9208024

[20] Gu S, Pasqualetti F, Cieslak M, et al. Controllability of structural brain networks. Nat Commun. 2015;6:8414. doi:10.1038/ncomms9414 26423222 PMC4600713

[21] Tozlu C, Card S, Jamison K, Gauthier SA, Kuceyeski A. Larger lesion volume in people with multiple sclerosis is associated with increased transition energies between brain states and decreased entropy of brain activity. Netw Neurosci. 2023;7(2):539-556. doi:10.1162/netn_a_00292 37397885 PMC10312270

[22] Broeders TAA, Schoonheim MM, Vink M, et al. Dorsal attention network centrality increases during recovery from acute stress exposure. Neuroimage Clin. 2021;31:102721. doi:10.1016/j.nicl.2021.102721 34134017 PMC8214139

[23] Schoonheim MM, Hulst HE, Brandt RB, et al. Thalamus structure and function determine severity of cognitive impairment in multiple sclerosis. Neurology. 2015;84(8):776-783. doi:10.1212/WNL.0000000000001285 25616483

[24] Steenwijk MD, Pouwels PJ, Daams M, et al. Accurate white matter lesion segmentation by k nearest neighbor classification with tissue type priors (kNN-TTPs). Neuroimage Clin. 2013;3:462-469. doi:10.1016/j.nicl.2013.10.003 24273728 PMC3830067

[25] Chard DT, Jackson JS, Miller DH, Wheeler-Kingshott CA. Reducing the impact of white matter lesions on automated measures of brain gray and white matter volumes. J Magn Reson Imaging. 2010;32(1):223-228. doi:10.1002/jmri.22214 20575080

[26] FSL 6. fmrib.ox.ac.uk/fsl.

[27] Pruim RHR, Mennes M, van Rooij D, Llera A, Buitelaar JK, Beckmann CF. ICA-AROMA: a robust ICA-based strategy for removing motion artifacts from fMRI data. Neuroimage. 2015;112:267-277. doi:10.1016/j.neuroimage.2015.02.064 25770991

[28] Cieslak M, Cook PA, He X, et al. QSIPrep: an integrative platform for preprocessing and reconstructing diffusion MRI data. Nat Methods. 2021;18(7):775-778. doi:10.1038/s41592-021-01185-5 34155395 PMC8596781

[29] Wang S, Peterson DJ, Gatenby JC, Li W, Grabowski TJ, Madhyastha TM. Evaluation of field map and nonlinear registration methods for correction of susceptibility artifacts in diffusion MRI. Front Neuroinform. 2017;11:17. doi:10.3389/fninf.2017.00017 28270762 PMC5318394

[30] Dhollander T, Mito R, Raffelt D, Connelly A. Improved white matter response function estimation for 3-tissue constrained spherical deconvolution. Proceedings of 27th International Society of Magnetic Resonance in Medicine; Montréal, Québec, Canada; 2019.

[31] Fischl B. FreeSurfer. Neuroimage. 2012;62(2):774-781. doi:10.1016/j.neuroimage.2012.01.021 22248573 PMC3685476

[32] Yeh FC, Wedeen VJ, Tseng WY. Generalized q-sampling imaging. IEEE Trans Med Imaging. 2010;29(9):1626-1635. doi:10.1109/TMI.2010.2045126 20304721

[33] Smith SM, Jenkinson M, Johansen-Berg H, et al. Tract-based spatial statistics: voxelwise analysis of multi-subject diffusion data. Neuroimage. 2006;31(4):1487-1505. doi:10.1016/j.neuroimage.2006.02.024 16624579

[34] Fan L, Li H, Zhuo J, et al. The human Brainnetome atlas: a new brain atlas based on connectional architecture. Cereb Cortex. 2016;26(8):3508-3526. doi:10.1093/cercor/bhw157 27230218 PMC4961028

[35] Yeo BT, Krienen FM, Sepulcre J, et al. The organization of the human cerebral cortex estimated by intrinsic functional connectivity. J Neurophysiol. 2011;106(3):1125-1165. doi:10.1152/jn.00338.2011 21653723 PMC3174820

[36] Smith RE, Tournier JD, Calamante F, Connelly A. Anatomically-constrained tractography: improved diffusion MRI streamlines tractography through effective use of anatomical information. Neuroimage. 2012;62(3):1924-1938. doi:10.1016/j.neuroimage.2012.06.005 22705374

[37] Smith R, Skoch A, Bajada CJ, Caspers S, Connelly A. Hybrid surface-volume segmentation for improved anatomically-constrained tractography. 2020.

[38] Smith RE, Tournier JD, Calamante F, Connelly A. SIFT2: enabling dense quantitative assessment of brain white matter connectivity using streamlines tractography. Neuroimage. 2015;119:338-351. doi:10.1016/j.neuroimage.2015.06.092 26163802

[39] Snyder W, Uddin LQ, Nomi JS. Dynamic functional connectivity profile of the salience network across the life span. Hum Brain Mapp. 2021;42(14):4740-4749. doi:10.1002/hbm.25581 34312945 PMC8410581

[40] GitHub. github.com/taabroeders/research-projects/tree/main/states_2024.

[41] Berman S, Backner Y, Krupnik R, et al. Conduction delays in the visual pathways of progressive multiple sclerosis patients covary with brain structure. Neuroimage. 2020;221:117204. doi:10.1016/j.neuroimage.2020.117204 32745679

[42] Sorrentino P, Pathak A, Ziaeemehr A, et al. The virtual multiple sclerosis patient: on the clinical-radiological paradox. medRxiv. 2023. doi:10.1101/2023.12.01.23299274

[43] Huiskamp M, Kiljan S, Kulik S, et al. Inhibitory synaptic loss drives network changes in multiple sclerosis: an ex vivo to in silico translational study. Mult Scler. 2022;28(13):2010-2019. doi:10.1177/13524585221125381 36189828 PMC9574900

[44] Sengupta B, Laughlin SB, Niven JE. Balanced excitatory and inhibitory synaptic currents promote efficient coding and metabolic efficiency. PLoS Comput Biol. 2013;9(10):e1003263. doi:10.1371/journal.pcbi.1003263 24098105 PMC3789774

[45] Huiskamp M, Yaqub M, van Lingen MR, et al. Cognitive performance in multiple sclerosis: what is the role of the gamma-aminobutyric acid system? Brain Commun. 2023;5(3):fcad140. doi:10.1093/braincomms/fcad140 37180993 PMC10174207

[46] He X, Caciagli L, Parkes L, et al. Uncovering the biological basis of control energy: structural and metabolic correlates of energy inefficiency in temporal lobe epilepsy. Sci Adv. 2022;8(45):eabn2293. doi:10.1126/sciadv.abn2293 36351015 PMC9645718

[47] Shine JM, Bissett PG, Bell PT, et al. The dynamics of functional brain networks: integrated network states during cognitive task performance. Neuron. 2016;92(2):544-554. doi:10.1016/j.neuron.2016.09.018 27693256 PMC5073034

[48] Koubiyr I, Broeders TAA, Deloire M, et al. Altered functional brain states predict cognitive decline 5 years after a clinically isolated syndrome. Mult Scler J. 2022;28(12):1973-1982. doi:10.1177/13524585221101470 35735004

[49] Filippi M, Krähenmann R, Fissler P. The link between energy-related sensations and metabolism: implications for treating fatigue. Front Psychol. 2022;13:920556. doi:10.3389/fpsyg.2022.920556 35800955 PMC9255916

[50] Fang F, Godlewska B, Cho RY, Savitz SI, Selvaraj S, Zhang Y. Personalizing repetitive transcranial magnetic stimulation for precision depression treatment based on functional brain network controllability and optimal control analysis. Neuroimage. 2022;260:119465. doi:10.1016/j.neuroimage.2022.119465 35835338

[51] Singleton SP, Luppi AI, Carhart-Harris RL, et al. Receptor-informed network control theory links LSD and psilocybin to a flattening of the brain's control energy landscape. Nat Commun. 2022;13(1):5812. doi:10.1038/s41467-022-33578-1 36192411 PMC9530221

[52] Finn ES, Bandettini PA. Movie-watching outperforms rest for functional connectivity-based prediction of behavior. Neuroimage. 2021;235:117963. doi:10.1016/j.neuroimage.2021.117963 33813007 PMC8204673

